# Comparative Transcriptome Analysis between a Novel Allohexaploid Cotton Progeny CMS Line LD6A and Its Maintainer Line LD6B

**DOI:** 10.3390/ijms20246127

**Published:** 2019-12-05

**Authors:** Jie Zheng, Xiangjun Kong, Bin Li, Aziz Khan, Zhiling Li, Yiding Liu, Haodong Kang, Farman Ullah Dawar, Ruiyang Zhou

**Affiliations:** 1 Key Laboratory of Plant Genetics and Breeding, College of Agriculture, Guangxi University, Nanning 530006, China; zhengjieself@163.com (J.Z.); kongxiangjun201010@163.com (X.K.); 18697998850@163.com (B.L.); aziz.hzau@gmail.com (A.K.); 18404968530@163.com (Z.L.); liuyiding1988@163.com (Y.L.); khd2414630992@163.com (H.K.); 2 Department of Zoology, Kohat University of Science and Technology, Kohat 26000, Pakistan; farmandawar@kust.edu.pk

**Keywords:** cotton, allohexaploid, cytoplasmic male sterility, transcriptome, differentially expressed genes

## Abstract

Cytoplasmic male sterility (CMS) is an important agronomic feature and provides an effective tool for heterosis utilization of crops. This study reports the comparative transcriptomic sketches between a novel allohexaploid cotton progeny CMS line LD6A and its maintainer line LD6B using de novo transcriptome sequencing technology at the pollen abortion stage. A total of 128,901 Unigenes were identified, in which 2007 were upregulated and 11,864 were downregulated. The significantly differentially expressed genes (DEGs) in LD6A show a distant and diverse genetic nature due to their distant hybrid hexaploidy progeny. Further analysis revealed that most of the DEGs participated in the tricarboxylic acid (TCA) cycle, oxidative phosphorylation, histone acetyltransferase activity, sepal development, stigma development, cotyledon development and microsporogenesis. A highly differentially expressed toxic protein, Abrin, was identified in the CMS line LD6A, which can catalyze the inactivation of ribosomes and consequently lead to cell death through the mitochondrial pathway in human cells. Twelve DEGs were selected randomly to validate transcriptome data using quantitative reverse-transcribed PCR (qRT-PCR). This study will contribute to new ideas and foundations related to the molecular mechanism of CMS and the innovation of cotton germplasm resources.

## 1. Introduction

Cotton is widely cultivated for fiber and oil seed production globally [1]. In China, cotton production has been improved by artificial emasculation and pollination, however this required a long time, intensive labor and cost to achieve [2]. Cytoplasmic male sterility (CMS) is a classical and convenient way to improve crop yields in cotton [3,4,5], kenaf [6], sweet orange (*Citrus sinensis*) [7], rape (*Brassica napus*) [8] and chili pepper (*Capsicum annuum* L.) [9]. Producing superior varieties using the hybridization method improves crop yields, however, interspecific hybrid incompatibility occurs in distant hybridization. Ancient natural allopolyploidization events and the superior properties of modern crop plants provides a driver of speciation and environmental adaptation. These methods of distant hybridization and allopolyploidization are the classical way to develop new germplasms [10]. In this study, a new CMS line LD6A was produced through the comprehensive utilization of distant hybridization, polyploidization and backcross, which is the first CMS germplasm that has been produced in this way in cotton. However, the molecular mechanism of CMS is still unclear.

In 1976, the carrier of the CMS factor was found to be mitochondrial DNA [11]; more than 50 mitochondrial genes were identified as CMS-relevant in various plants [12,13,14,15]. Several metabolic pathways which influence biological processes that cause CMS via the action of mitochondrial genes coupled with nuclear genes were identified [16]. Studies have been done regarding global transcriptional networks from big data analysis to explore CMS genes, which have been shown to be associated with the tricarboxylic acid (TCA) cycle, respiratory electron transport chain, oxidative phosphorylation and toxic proteins [6,17,18,19,20].

In the backcrossing process, progeny of distant hybridization with chromosomes that come from wild cotton could not be completely replaced [21]. Moreover, the genome sequences of the allohexaploid progeny of *Gossypium raimondii* (DD) [22], *Gossypium arboreum* (AA) [23], and *Gossypium hirsutum* (AADD) [24] revealed unclear gene annotation. The single reference genome of diploid and tetraploid cotton could not completely restore the basic features of the transcriptome; rather, the de novo assembly method could annotate the transcriptomic data.

However, limited sources of the CMS line and its negative effects on the cytoplasm inhibited cotton yield. In this context, identification of the molecular mechanism and development of new CMS germplasm resources are essential.

To understand the molecular mechanism of LD6A pollen abortion, we analyzed the gene expression at the tetrad stage (abortion stage) in a novel CMS line and its maintainer line on a global scale using a de novo assembly transcriptome. Our study reveals that Abrin, BTB/POZ and TAZ family genes, MDH, WRKY40, and *atp8* were closely related to CMS. Our findings are interesting and facilitate understanding of the CMS molecular mechanism, which will provide further knowledge for germplasm innovation and heterosis utilization.

## 2. Results

### 2.1. De Novo Transcriptome Analysis

**Figure 1:**
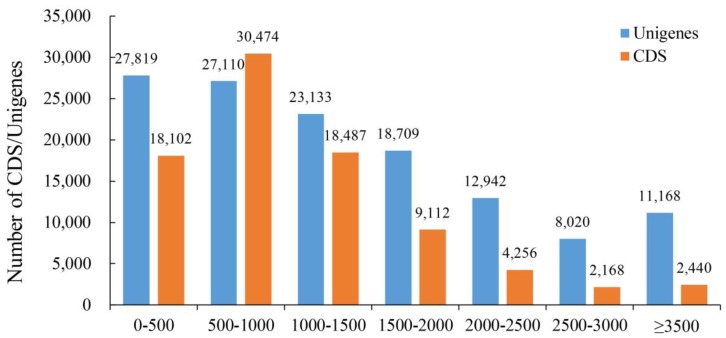
Length distribution of the assembled cotton genes and CDS. The horizontal axis shows the size of the Unigenes/CDS and the vertical axis shows the number of Unigenes/CDS.

In this study, the transcriptome sequencing of the CMS lines LD6A and LD6B at the tetrad stage was conducted using an Illumina Hi-Seq 4000. A total of 39.57 gigabytes of raw data (73.88 million raw reads) was obtained from six libraries. After filtering, 65.96 million clean reads were identified. The de novo profiles were performed with all clean reads using Trinity software (v2.4.0) (Table 1). After assembling, a total of 709,971 transcripts were identified, which has 680,840,189 bases (Table 1). The N50 statistics showed that more than 50% of transcripts were longer than 1541 bp. The N50 of all genes was 2036 bp, while the average length of all genes was 1434 bp (Table 1). The length distribution of all assembled cotton genes is shown in Figure 1, which shows that 24.9% of the total Unigenes and 10.4% of the total CDS were longer than 2000 bp.

### 2.2. Annotation of the Assembled Cotton Genes

**Figure 2:**
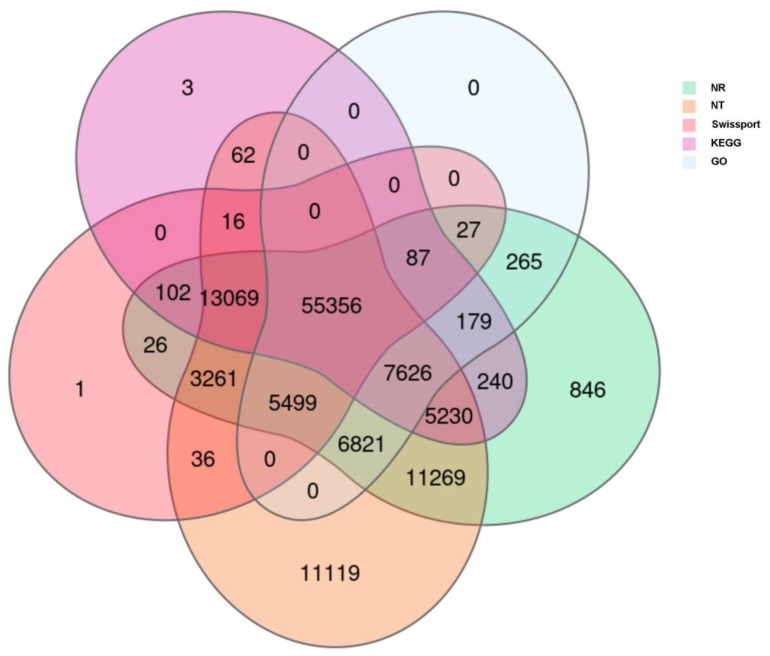
Venn diagram of genes identified to different databases.

**Figure 3:**
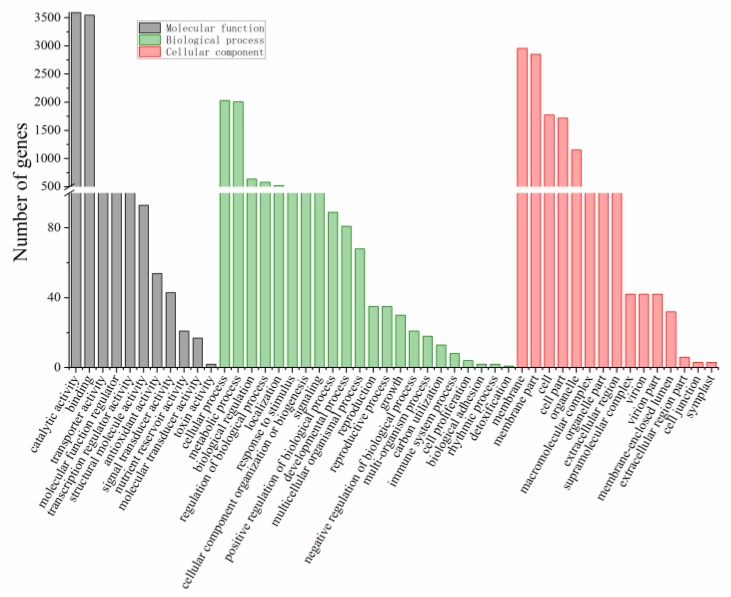
Gene ontology classification of the assembled cotton genes.

All sequences were aligned against public databases: 109,903 (NR: 85.26%), 119,364 (NT: 92.60%), 77,480 (SwissProt: 60.11%), 83,201 (KOG: 64.55%), 81,970 (KEGG: 63.59%), 75,860 (GO: 58.85%) and 71,567 (Pfam: 55.52%) for functional annotation (Figure 2). GO annotation shows that 17,037, 4416 and 33,804 genes were involved in cellular process, metabolic process and catalytic activity, respectively (Figure 3). Furthermore, genes responsible for antioxidant activity (398 genes), toxin activity (8 genes) and response to stimuli (2035 genes) were identified in this study (Figure 3). The top five KEGG pathways were “global and overview maps” (18,738 genes), “carbohydrate metabolism” (7371 genes), “translation” (6271 genes), “folding, sorting and degradation” (6155 genes), and “signal transduction” (5554 genes). The Unigene sequences were annotated into the KOG database to obtain classification information of gene homologies. “General function prediction only” (18,372 genes), “signal transduction mechanisms” (9774 genes), “function unknown” (7574 genes), “post-translational modification, protein turnover, chaperones” (6504 genes) and "transcription” (5680 genes) were the top five classes. Multiple annotation perspectives of the assembled cotton transcriptome were performed to deeply understand the differences in microsporogenesis and the physiological and biochemical characteristics between the CMS line and its maintainer line. In these annotations, 85,312 CDS were detected by TransDecoder (https://transdecoder.github.io). At the same time, 29,807 SSRs were detected in 23,774 Unigenes, among which 5141 Unigenes encoding transcription factors (TF) were predicted. Meanwhile, 9537 out of 128,901 Unigenes were predicted to encode proteins that have not been annotated.

### 2.3. Annotation Difference Analysis of the Allohexaploid Progeny of Cotton

**Figure 4:**
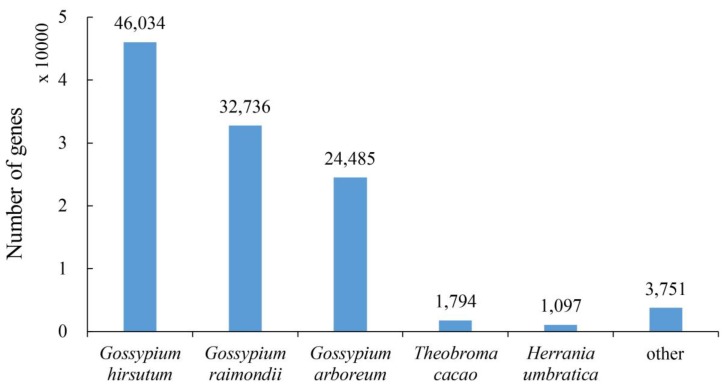
Distribution of species aligned by the assembled cotton.

**Figure 5:**
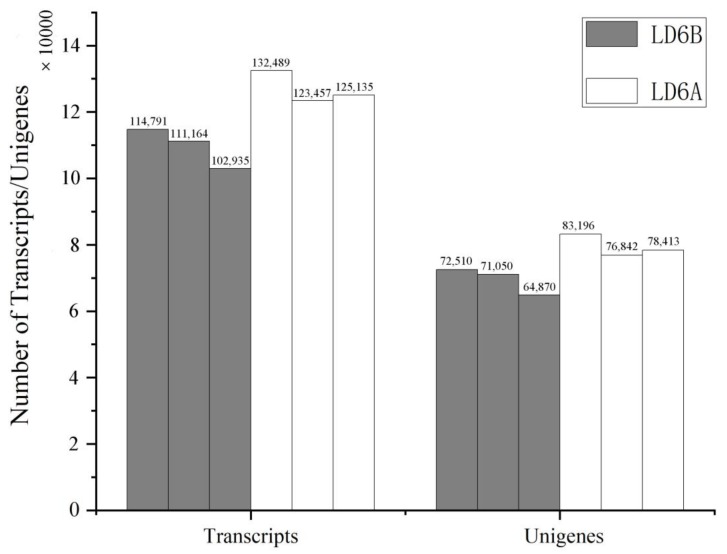
Number of transcripts and Unigenes annotated in different samples.

The distribution of species aligned by the assembled allohexaploid progeny of cotton shows that 46,034 genes were from *Gossypium hirsutum*, 32,736 from *Gossypium raimondii*, 24,485 from *Gossypium arboretum*, 1794 from *Theobroma cacao*, 1097 from *Herrania umbratica* and 3751 were found from other species (Figure 4). The CMS line showed more annotated transcripts and genes compared to the maintainer line (Figure 5). 

### 2.4. Analysis of Differentially Expressed Genes (DEGs)

**Figure 6:**
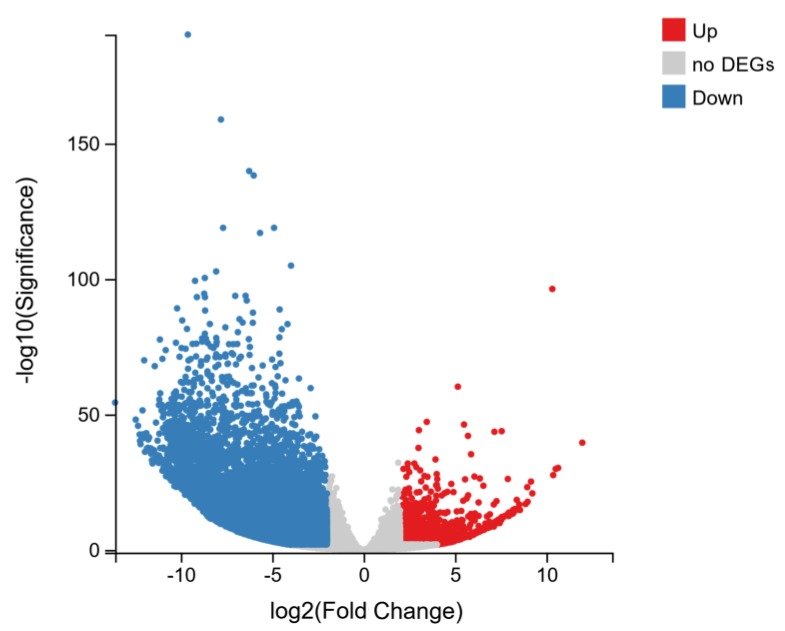
Comparison of gene expression levels between LD6A and LD6B according to the DEseq2 method. DEGs = differentially expressed genes.

**Figure 7:**
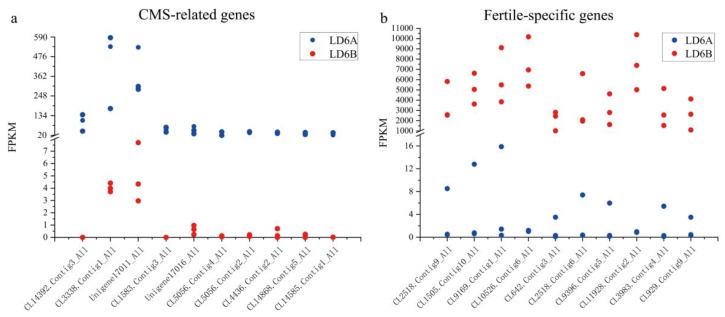
(**a**) Indicates highly expressed genes exclusively identified in LD6A. (**b**) Top 10 highly expressed genes identified exclusively in LD6B samples. FPKM = fragments per kilobase of transcript per million fragments mapped.

Three biological replicates of both the CMS and maintainer line were pooled using the DEseq2 method (fold change ≥2.00 and adjusted *p* value ≤ 0.05), in which 13,871 DEGs were obtained from LD6A vs. LD6B (Figure 6, Supplementary Materials 1). Among the DEGs, the top 10 upregulated CMS-related genes and top 10 downregulated fertile-specific genes were detected. These highly upregulated genes encoded nitrate or di/tri-peptide transporters NRT1/PTR FAMILY 3.1 (CL14392.Contig3_All), non-specific lipid-transfer protein A (CL3338.Contig1_All), repetitive proline-rich cell wall protein 1 (Unigene17011_All), repetitive proline-rich cell wall protein 2-like (Unigene17016_All), ATP synthase protein YMF19 (CL1583.Contig3_All), zinc finger protein constants-like 16 (CL5056.Contig4_All, CL5056.Contig2_All), cup-sharp cotyledon3 (CL4436.Contig2_All) and an uncharacterized protein (CL14868.Contig5_All, CL14585.Contig1_All) which are associated to CMS (Figure 7a). The top 10 genes (Figure 7b) that were identified in the maintainer line and downregulated in the CMS line that may be related to fertility encode polygalacturonase (CL2518.Contig9_All, CL2518.Contig6_All), pectinesterase (CL1505.Contig10_All), Parus major synaptotagmin 11 (CL9169.Contig1_All), transcript variant X1 arabinogalactan peptide 23 (CL10526.Contig6_All), non-specific lipid-transfer protein 2 (CL642.Contig3_All), uncharacterized ncRNA (CL9396.Contig5_All), L-ascorbate oxidase homolog (CL11928.Contig2_All), putative pectinesterase 63 (CL3983.Contig4_All), and pollen allergen Che a 1 (CL929.Contig9_All). In addition, differentially expressed genes were also identified that encode various protein products, including the toxic protein Abrin, transcription factors BTB/POZ and TAZ, cytoplasmic malate dehydrogenase, stress-induced transcription factors (such as WRKY40 which influences toxic activity), histone acetyltransferase, components of the tricarboxylic acid (TCA) cycle and the development of sepals, stigmas and cotyledons, respectively.

### 2.5. GO Annotation and Pathway Analysis of DEGs

To better understand the relationship between DEGs and CMS, GO classification and functional enrichment were carried out to perform the functional analysis of DEGs (Supplementary Materials 2–4). “Catalytic activity”, “binding”, “membrane”, “membrane part” and “cellular process” were the top five terms among the 48 GO terms classified from 7648 DEGS. Most of the DEGs were allied with catalytic activity, binding, transporter activity and molecular function regulator.

Moreover, among the significant GO terms (Table 2), 2742, 198, and 144 genes were involved in “integral component of membrane” (GO:0016021), “carbohydrate metabolic process” (GO:0005975) and “extracellular region” (GO:0005576), respectively. Kyoto Encyclopedia of Genes and Genomes (KEGG) pathway classification and functional enrichment of DEGs was used to identify metabolic networks and biological pathways. Furthermore, 4500 DEGs were annotated and classified into 133 groups and 27 KEGG pathways were significantly enriched with *p* ≤ 0.05 and Q ≤ 0.05 (Table 3). The 285 DEGs were involved in endocytosis (ko04144), starch and sucrose metabolism (262, ko00500), amino sugar and nucleotide sugar metabolism (230, ko00520), pentose and glucuronate interconversions (224, ko00040) and phenylpranoid biosynthesis (209, ko00940).

### 2.6. Validation of DEGs by qRT-PCR

qRT-PCR was used to verify the reliability of RNA sequencing (Illumina sequencing). Twelve DEGs (six upregulated and six downregulated) were selected randomly. The results indicated that the Illumina sequencing data were reliable (Table 4). The fold change of some DEGs confirmed by qRT-PCR was different to the fold change detected by RNA-Seq, which may be due to the different computing methods of the two technologies: RNA-Seq calculates absolute quantification, whereas qRT-PCR detect relative expression.

### 2.7. Candidate Genes Associated with CMS

We further analyzed the DEGs in different categories that might be related to CMS, toxic proteins, HATs (histone acetyltransferase) related proteins, TCA cycle related proteins, transcription factors, and some other proteins [6,17,18,19,20].

#### 2.7.1. Abrin

**Figure 8:**
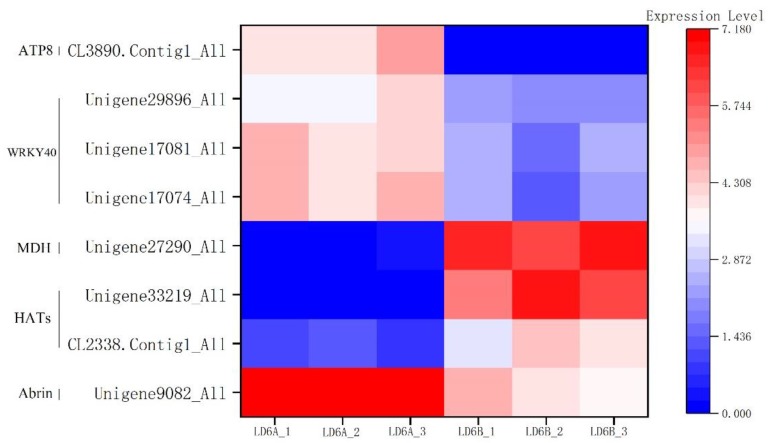
The heat map of key candidate genes associated with CMS.

In this study, eight genes were identified that encoded proteins with toxic activity. Interestingly, one gene (Unigene9082) was differentially expressed (Figure 8) and was significantly upregulated in the CMS line compared with the maintainer line. After the sequence was aligned against NCBI Blast (https://blast.ncbi.nlm.nih.gov/Blast.cgi), it was predicted to be a toxic protein called Abrin that can be found in cotton. 

#### 2.7.2. Histone Acetyltransferase (HATs)

Differential gene expression analysis revealed that two genes (Unigene33219 and CL2338.Contig1) were associated with histone acetyltransferase. These genes were significantly downregulated in the CMS line (Figure 8). 

#### 2.7.3. TCA Cycle

Gene expression analysis showed a significantly differentially expressed gene called Unigene27290 (Figure 8). After alignment to NCBI, it was predicted to be a cytoplasmic malate dehydrogenase (MDH) gene. Molecular identification and characterization also showed it to be malate dehydrogenase (MDH, EC 1.1.1.37), which belongs to the class A dehydrogenase family. This can form a highly conserved NAD (P) ~+ dependent gene family and can catalyze the reversible conversion of oxaloacetate and malate.

#### 2.7.4. WRKY40

GO analysis of genes retrieved from sepal, stigma and cotyledon development revealed that three genes (Unigene29896, unigene17074 and unigene17081) were highly differentially expressed (Figure 8). All encoded a transcription factor family called WRKY40, a stress-inducible transcription factor gene that plays an important role in stress.

#### 2.7.5. Oxidative Phosphorylation

There were 45 DEGs associated with oxidative phosphorylation, including six upregulated and 39 downregulated. Oxidative phosphorylation is the most important process of energy metabolism and closely related to organ development and microspore development. Inadequate energy supply during microspore development causing microspore abortion is the main cause of CMS. Interestingly, an upregulated gene encodes a subunit of the ATP synthase (Figure 8), a mitochondrial gene called atp8, which plays an important role in the respiratory chain.

#### 2.7.6. Transcription Factors

**Figure 9:**
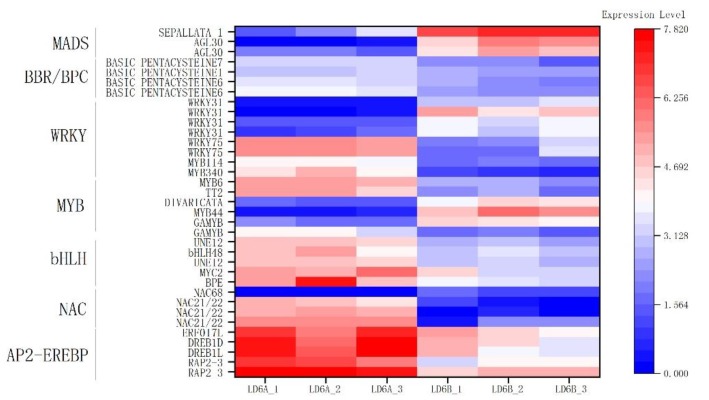
The heatmap of key transcription factor family genes related to CMS.

We found 5141 cotton genes with potential of encoding TFs, falling into 59 categories. We detected AP2-EREBP, NAC, bHLH, MYB, WRKY, MADS, BBR/BPC to analyze gene expression, respectively (Figure 9). These selected transcription factors are associated with biological development, energy metabolism, toxic activity protein and other functions.

## 3. Discussion

In this study, RNA sequencing technology was used to explore gene expression profiles of cotton associated with CMS compared to a maintainer line. The CMS line was obtained from the progeny of an allohexaploid cotton produced by distant hybridization of hexaploidy wild cotton and tetraploid upland cotton. The isogenic lines were produced by backcrossing with maintainer lines for many generations. Therefore, the CMS line LD6A is more suitable for studying the molecular mechanism of CMS and chromosome evolution.

Traditionally, near-isogenic lines are similar at the nuclear gene level, however the current transcriptomic study showed that their gene expression levels are quite different (13,871 DEGs out of 128,901 assembled genes), which indicated that some genetic material from the wild cotton (*Gossypium stocksii*) could not be totally replaced in the backcross process [21]. It plays an important role in gene expression and may be the key factor for microspore abortion in the CMS line (LD6A). Previously, sources of the CMS and maintainer line used for comparative analysis were quite different, even compared with the other near-isogenic lines [6,17]. Since the first cotton reference genome was published [22], more assembly reference genomes of diploid and tetraploid cotton have been developed [23,24,25,26] and the evolution of the cotton genome has been more focused [27,28]. However, little is known about the chromosome background of allohexaploid cotton. In this context, the current de novo assembly method was necessary, which annotated the full transcriptome data of LD6A.

The CMS line and its restoration system are the main pollination control systems for hybrid production by crop heterosis [29]. However, limited CMS and restorer germplasm resources, lack of effective pollination media and photo-temperature sensitivity of the restorer system prevent the increase of cotton yield. Identification of the molecular mechanism and development of new CMS germplasm resources are vital.

Histone acetyltransferases (HATs) play an important role in the structural modification of chromosomes and regulation of gene expression [30]. HATs and HDAs (histone deacetylases) are emerging as important components of protein complexes that affect the dynamics of chromatin folding during gene transcription [31,32]. HATs and HDAs are co-regulated to keep a dynamic balance between histone acetylation and histone deacetylation. A BTB/POZ and TAZ domain-containing protein with HAT activity was identified, which can affect the function and stability of many proteins. In the process of polyploidization and backcrossing, this balance may be destroyed by the differential expression of HATs, which may cause CMS.

During microspore development, an inadequate energy supply and reactive oxygen species (ROS) play a key role leading to pollen abortion. This energy is supplied by the mitochondria, which are the core sites for energy metabolism [33]. Therefore, we focused on the DEGs related to energy metabolism and biological development. A cytoplasmic malate dehydrogenase (MDH) gene related to the TCA cycle was found to be significantly downregulated in the CMS line, located in the cytoplasm. This participated in many metabolic pathways such as carbohydrate metabolism and lipid metabolism [34]. It has many biological functions in the glyoxylic acid cycle, tricarboxylic acid cycle, glucose synthesis, amino acid synthesis and redox stability [35]. In addition, malate dehydrogenase is a plant stress resistance and a candidate gene to breed cotton cultivars for increasing insoluble P absorption [36].

Meanwhile, a mitochondrial gene called *atp8*, related to oxidative phosphorylation, was upregulated in the present study but downregulated in UG93 [6,37]. This indicates that excessive ATP production and inadequate ATP consumption may lead to reactive oxygen species (ROS) explosion, which may cause cell toxicity and microspore abortion.

In this study, a toxic protein called Abrin was identified, which can cause mitochondrial apoptosis via the ribosomal pathway in human cells [38]. Toxic protein production is considered to be the cause of CMS [39]. The Database of Interacting Proteins (DIP) showed two proteins that interact with Abrin. One is glycosylation related, UDP-N-acetyl-D-galactosamine: polypeptide N-acetylgalactosaminyltransferase (Gly5, DIP: 26207N), which is located on the Golgi apparatus. Another is acetylglutamate kinase (NAGK, DIP: 5348N), which is a catalytic enzyme for the second step of arginine biosynthesis located in plastids and regulates gametophytic function and embryonic development [40]. This provides a new direction for studying the molecular mechanism of CMS and mitochondrial programmed cell death. Further molecular based research is recommended to explore the function of Abrin in cotton.

Transcription factors (TFs) play several roles in plant physiology throughout life. AP2-EREBP is a key regulator of several developmental processes, such as floral organ identity determination, to form part of the mechanisms used by plants to respond to various types of biotic and environmental stress [41]. NAC transcription factors interact directly or indirectly with other proteins by binding to DNA, participating in the plant biological and abiotic stress response, hormone signaling pathway transduction, apoptosis and other processes [42]. bHLH transcription factors are essential for the normal growth and development of plants, and act against various abiotic stresses in plants [43]. The MYB transcription factor is widely involved in plant metabolic regulation [44]. The WRKY gene family plays essential roles in diverse stress responses, and developmental and physiological processes [45]. A transcription factor family protein called WRKY40 is related to sepal, stigma and cotyledon development. It acts as a a stress-inducible transcription factor gene and plays an important role in stress. We speculated that WRKY40 may be related to the accumulated ROS stress response. MADS transcription factors play an important role in flower development [46]. BBR/BPC transcription factors respond to ethylene with DNA-binding transcription factor activity and sequence-specific DNA binding [47]. The relationship between CMS and TFs with abnormal transcription level needs further study.

## 4. Materials and Methods

### 4.1. Plant Materials

**Figure 10:**
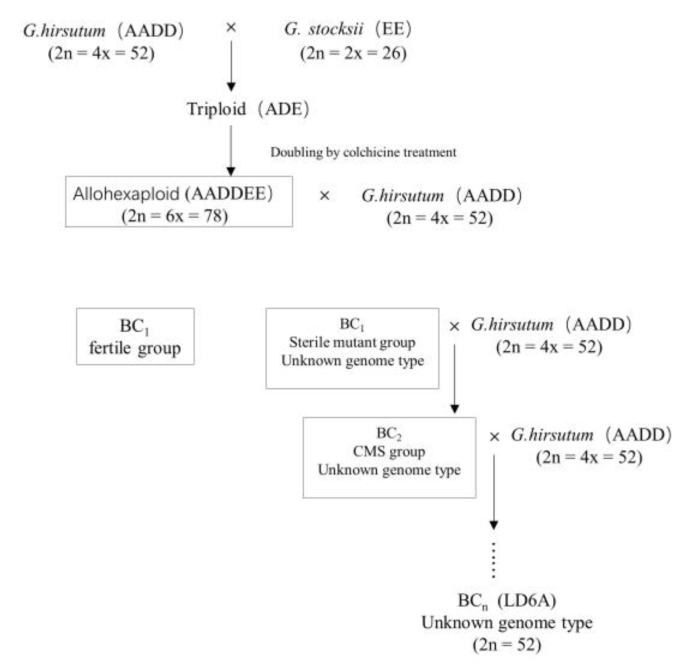
Diagrammatic view of the allohexaploid and backcrossed (BC) generations development procedure. CMS = cytoplasmic male sterility.

The CMS line is a progeny, which was developed by distant hybridization and the allopolyploidization method. Its parents, Zhongmian16 (*Gossypium hirsutum*, tetraploid, AADD, 2n = 52) and a wild cotton (*Gossypium stocksii*, diploid, EE, 2n = 26) provided distant hybridization that yielded a triploid branch (provided by the National Wild Cotton Nursery). To obtain a double genome, we treated the branch with 0.1% colchicine solution. After self-pollination, hexaploidy seeds were obtained and backcrossed with Zhongmian16, which yielded a CMS mutant progeny. Thereafter, the CMS mutant was backcrossed with Zhongmian16 (9 generations and, named LD6A. Zhongmian16 was named LD6B as its maintainer line (Figure 10).

Cotton were sown (April–October) at the experimental farm of Guangxi University (Nanning, summer) and the National Wild Cotton Nursery (Sanya, winter). After microscopic study, floral buds were collected from LD6A and LD6B. The pollen abortion stage (tetrad stage, 4–5 mm in diameter) were frozen in liquid nitrogen and stored at −80 °C for RNA isolation.

### 4.2. RNA Extraction, cDNA Library Construction and Sequencing

Total RNA of both LD6A and LD6B lines were extracted from the floral bud (tetrad stage) using an RNA Isolation Kit (TransGen Biotech, Beijing, China). From each sample, 3 µg of the total RNA (RIN ≥8) was used for transcriptome cDNA library construction with a TruSeq™ RNA Sample Preparation Kit v2 (Illumina, San Diego, CA, USA). RNA purification beads with oligo (dT) were used to separate mRNA from the total RNA. After breaking the mRNA into short fragments in fragmentation buffer, double-stranded cDNA was synthesized (Super ScriptII reverse transcriptase, Invitrogen, Carlsbad, CA, USA) and purified (Agencourt AMPure XP-Medium, Agencourt, Carlsbad, CA, USA). The short cDNA fragments were end-repaired with an A-tail addition and connected with adapters. After agarose gel electrophoresis, suitable fragments were used as templates for PCR amplification. The Agilent 2100 Bioanalyzer (Agilent, Santa Clara, CA, USA) and an ABI StepOnePlus Real-Time PCR System were respectively used for quantification and qualification of the sample cDNA library. Solexa sequencing was performed by BGI (Shenzhen, China) using an Illumina HiSeq 4000 platform. The raw sequencing files of these six samples (FASTQ files) are accessible from the NCBI Sequence Read Archive (SRA) database under Accession Number PRJNA577562.

### 4.3. De Novo Transcriptome Assembly and Gene Expression Profile

Raw sequence data were processed to obtain clean reads by SOAPnuke (self-developed by BGI, version: v1.4.0) and trimmomatic (version: v0.36) for filtering low-quality reads (quality score <20). Trinity (version v2.0.6) was used for de novo assembly, and then Tgicl (https://github.com/trinityrnaseq/trinityrnaseq/wiki) was used to cluster the assembled transcripts in Unigene. All coding sequences were predicted from Unigene using TransDecoder (version v3.0.1, https://transdecoder.github.io). Unigene was detected for SSR sequences by MISA (version v1.0, http://pgrc.ipk-gatersleben.de/misa), and primers were designed by Primer3 (version v2.2.2, http://bioinfo.ut.ee/primer3). High-quality clean reads were mapped with reference gene sequences by Bowtie2 (v2.2.5, http://bowtie-bio.sourceforge.net/Bowtie2/index.shtml). The expression levels of genes and transcripts were calculated by RSEM (version: v1.2.8 http://deweylab.biostat.wisc.edu/rsem/rsem-calculate-expression.html).

### 4.4. Gene Functional Annotation Analysis

For gene functional annotation, data from Unigene were aligned against various databases (KEGG, http://www.genome.jp/kegg; GO, http://geneontology.org; NR, ftp://ftp.ncbi.nlm.nih.gov/blast/db; NT, ftp://ftp.ncbi.nlm.nih.gov/blast/db; Pfam, http://pfam.xfam.org; SwissProt, https://www.uniprot.org; KOG, ftp://ftp.ncbi.nih.gov/pub/COG/KOG/kyva) using a combination of BLAST (http://blast.ncbi.nlm.nih.gov/Blast.cgi), HMMER (http://hmmer.janelia.org/), Blast2GO (http://www.blast2go.com/b2ghome), and KEGG Automatic Annotation Server (KAAS; http://www.genome.jp/kegg/kaas/). According to the results of NR annotation, the proportion of different species on the annotation was counted and the species distribution figure was drawn.

### 4.5. Transcription Factor Analysis

In order to determine plant transcription factors, the ORF in Unigene was detected using getorf (version EMBOSS:6.5.7.0, http://emboss.sourceforge.net/apps/cvs/emboss/apps/getorf.html). Further, hmmsearch (version v3.0, http://hmmer.org) was used to compare ORFs with the domain of transcription factor proteins (data from TF). The previous two steps enabled us to identify the ability of Unigene to identify the characteristics of the transcription factor family described by the plant TFDB (http://plntfdb.bio.uni-potsdam.de/v3.0/).

### 4.6. Individualized Bioinformatics Analysis

All clean data were analyzed by Dr. Tom, an online software developed by BGI (http://report.bgi.com). Cluster heatmapping, GO enrichment and KEGG enrichment were carried out, followed by standard process.

### 4.7. Verification of Gene Expression by qRT-PCR

The relative expression of the DEGs was verified by qRT-PCR and analyzed using the 2^-∆∆*C*t^ method [48], where the 18s gene was considered as the endogenous control. All the primers were designed by Primier 5.0 (Supplementary Materials 5) and synthesized by BGI (Shenzhen, China). RT-PCR and qRT-PCR were performed according to the method previously used [17].

## 5. Conclusions

In this study, the transcriptomes of the allohexaploid progeny cotton CMS line LD6A and its maintainer line LD6B were investigated using detailed RNA sequencing methodology. Thousands of DEGs were assessed between LD6A and LD6B focusing on their key biological processes and energy metabolism. The toxic protein, Abrin, and several other key DEGs such as BTB/POZ and TAZ family genes, MDH, WRKY40 and *atp8* were found to be closely related to CMS. These genes are mainly involved in the TCA cycle, respiratory electron transfer chain, and oxidative phosphorylation, which were considered to be candidate CMS genes. Our findings will improve understanding of the gene regulation mechanism in CMS and the evolution of chromosomes.

## Figures and Tables

**Table 1:** Overview of the assembled cotton transcriptome. Mb = megabytes.

Type	LD6B		LD6A
Total raw reads	73.88 Mb		73.88 Mb
Total trinity transcripts		709,971	
N50 (transcripts)		1541	
Total assembled bases (transcripts)		680,840,189	
Total trinity genes		128,901	
N50 (genes)		2036	
Average length (genes)		1434	
Total assembled bases (genes)		184,861,957	
GC (%)		40.66	

**Table 2:** GO enrichment analysis of DEGs in the LD6A vs. LD6B library.

Type	ID	Term	Number	Rich Ratio	*p* Value
Biological process	GO:0045490	pectin catabolic process	89	0.028507367	1.52 × 10^−39^
	GO:0042545	cell wall modification	61	0.019538757	4.79 × 10^−26^
	GO:0005975	carbohydrate metabolic process	198	0.063420884	3.23 × 10^−16^
	GO:0071555	cell wall organization	108	0.034593209	3.49 × 10^−10^
	GO:0016042	lipid catabolic process	57	0.018257527	3.77 × 10^−9^
	GO:0051017	actin filament bundle assembly	13	0.004163997	1.60 × 10^−8^
Cellular component	GO:0016021	integral component of membrane	2742	0.637377964	2.30 × 10^−29^
	GO:0005576	extracellular region	144	0.033472803	3.17 × 10^−23^
	GO:0005618	cell wall	92	0.021385402	5.67 × 10^−10^
Molecular function	GO:0004857	enzyme inhibitor activity	69	0.011551984	1.53 × 10^−28^
	GO:0030599	pectinesterase activity	61	0.010212623	5.75 × 10^−26^
	GO:0045330	aspartyl esterase activity	61	0.010212623	5.75 × 10^−26^
	GO:0004650	polygalacturonase activity	54	0.009040683	1.65 × ^10−20^
	GO:0030570	pectate lyase activity	32	0.005357442	6.03 × 10^−17^
	GO:0005096	GTPase activator activity	59	0.009877783	2.81 × 10^−14^
	GO:0003779	actin binding	63	0.010547464	5.97 × 10^−11^
	GO:0045735	nutrient reservoir activity	21	0.003515821	7.83 × 10^−11^
	GO:0004575	sucrose alpha-glucosidase activity	14	0.002343881	1.11 × 10^−9^
	GO:0015299	solute: proton antiporter activity	34	0.005692282	2.59 × 10^−9^
	GO:0020037	heme binding	132	0.022099448	7.05 × 10^−8^

**Table 3:** Statistical enrichment analysis of Kyoto Encyclopedia of Genes and Genomes (KEGG) metabolic pathways (*p* ≤ 0.05, Q ≤ 0.05).

Pathway	Pathway ID	Genes with Pathway Annotation		*p* Value	Q Value
		DEGs (2934)	All Genes (19,296)		
Biosynthesis of other secondary metabolites					
Flavone and flavonol biosynthesis	ko00944	8	28	5.749022 × 10^−3^	2.874511 × 10^−2^
Stilbenoid, diarylheptanoid and gingerol biosynthesis	ko00945	27	150	2.693941 × 10^−3^	1.454728 × 10^−2^
Phenylpropanoid biosynthesis	ko00940	209	1613	2.371287 × 10^−4^	1.684862 × 10^−3^
Carbohydrate metabolism					
Glycolysis/gluconeogenesis	ko00010	119	902	2.607198 × 10^−3^	1.454728 × 10^−2^
Inositol phosphate metabolism	ko00562	98	709	1.457824 × 10^−3^	8.945738 × 10^−3^
Starch and sucrose metabolism	ko00500	262	2022	4.067709 × 10^−5^	3.922434 × 10^−4^
Galactose metabolism	ko00052	134	896	5.676684 × 10^−6^	8.515026 × 10^−5^
Amino sugar and nucleotide sugar metabolism	ko00520	230	1532	2.096337 × 10^−9^	7.075137 × 10^−8^
Pentose and glucuronate interconversions	ko00040	224	993	2.589022 × 10^−30^	3.495180 × 10^−28^
Digestive system					
Cholesterol metabolism	ko04979	26	122	2.363962 × 10^−4^	1.684862 × 10^−3^
Energy metabolism					
Carbon fixation in photosynthetic organisms	ko00710	71	485	1.431960 × 10^−3^	8.945738 × 10^−3^
Oxidative phosphorylation	ko00190	131	930	1.187092 × 10^−4^	1.001609 × 10^−3^
Photosynthesis	ko00195	39	189	1.710027 × 10^−5^	2.098670 × 10^−4^
Lipid metabolism					
Glycerophospholipid metabolism	ko00564	134	992	5.903167 × 10^−4^	3.984638 × 10^−3^
Linoleic acid metabolism	ko00591	30	143	1.108358 × 10^−4^	9.975222 × 10^−4^
Ether lipid metabolism	ko00565	61	351	3.005129 × 10^−5^	3.120711 × 10^−4^
Arachidonic acid metabolism	ko00590	37	178	2.341729 × 10^−5^	2.634445 × 10^−4^
Glycerolipid metabolism	ko00561	122	814	1.313738 × 10^−5^	1.773546 × 10^−4^
Steroid biosynthesis	ko00100	51	216	1.062244 × 10^−8^	2.390049 × 10^−7^
Cutin, suberine and wax biosynthesis	ko00073	59	260	3.884301 × 10^−9^	1.048761 × 10^−7^
Metabolism of other amino acids					
Cyan amino acid metabolism	ko00460	136	986	2.254576 × 10^−4^	1.684862 × 10^−3^
Diterpenoid biosynthesis	ko00904	36	236	1.022848 × 10^−2^	4.931589 × 10^−2^
Monoterpenoid biosynthesis	ko00902	24	135	5.264254 × 10^−3^	2.733363 × 10^−2^
Signal transduction					
Phosphatidylinositol signaling system	ko04070	104	770	2.261160 × 10^−3^	1.327203 × 10^−2^
Transcription					
RNA polymerase	ko03020	166	795	3.529257 × 10^−19^	1.588166 × 10^−17^
Transport and catabolism					
Endocytosis	ko04144	285	2152	3.517584 × 10^−6^	5.935923 × 10^−5^
Phagosome	ko04145	111	697	2.018673 × 10^−6^	3.893155 × 10^−5^

**Table 4:** qRT-PCR confirmation of the expression profiles of selected genes.

Gene ID	Protein Identity	Fold Change	
		RNA-Seq	qRT-PCR
LOC107915747	cytochrome P450 83B1-like	5.16	4.40
LOC107892026	zinc finger protein CONSTANS-LIKE 16-like	6.06	5.83
LOC107903815	methyltransferase-like protein	5.21	1.5
LOC107893683	mitochondrial uncoupling protein 3-like	5.56	3.13
LOC107911279	transcription factor MYB114-like	5.04	5.03
LOC107926337	probable calcium-binding protein CML49	6.69	0.19
LOC107905948	probable calcium-binding protein CML13	−10.76	−2.74
LOC107941623	pollen allergen Che a 1-like	−12.94	−26.59
LOC107942901	pollen-specific protein-like At4g18596	−11.48	−8.12
LOC107915309	plasma membrane ATPase 4-like	−10.61	−1.30
LOC107908343	V-type proton ATPase subunit G1-like (ATP6V1G1)	−10.45	−4.71
LOC107903454	cytochrome P450 76A2-like	−10.59	−4.71

## Supplementary Materials

The following are available online at https://www.mdpi.com/1422-0067/20/24/6127/s1.
